# Clinical Significance of microRNAs in Hematologic Malignancies and Hematopoietic Stem Cell Transplantation

**DOI:** 10.3390/cancers15092658

**Published:** 2023-05-08

**Authors:** Aneta Sevcikova, Ivana Fridrichova, Nataliia Nikolaieva, Lenka Kalinkova, Radoslav Omelka, Monika Martiniakova, Sona Ciernikova

**Affiliations:** 1 Department of Genetics, Cancer Research Institute, Biomedical Research Center of Slovak Academy of Sciences, 845 05 Bratislava, Slovakia; 2 Department of Botany and Genetics, Faculty of Natural Sciences and Informatics, Constantine the Philosopher University in Nitra, 949 74 Nitra, Slovakia; 3 Department of Zoology and Anthropology, Faculty of Natural Sciences and Informatics, Constantine the Philosopher University in Nitra, 949 74 Nitra, Slovakia

**Keywords:** microRNA, hematologic malignancies, malignant hematopoiesis, hematopoietic stem cell transplantation, graft-versus-host disease, miRNA-based approach

## Abstract

**Simple Summary:**

Dysregulated microRNA (miRNA) expression has been described in the initiation and progression of a wide spectrum of hematologic malignancies. Moreover, aberrant miRNA expression patterns have been shown to be involved in severe post-transplant complications in patients receiving hematopoietic stem cell transplantations (HSCT), suggesting their use as promising diagnostic and prognostic biomarkers for acute graft-versus-host disease (aGvHD). Recently, activation or inhibition of deregulated miRNAs appears to be an attractive therapeutic option for increasing treatment efficacy. Further research concerning the associations between alterations in miRNA levels and malignant hematopoiesis and treatment response might bring new possibilities for specific miRNA-based approaches to improve the outcomes for hematologic cancer patients.

**Abstract:**

Hematologic malignancies are a group of neoplastic conditions that can develop from any stage of the hematopoiesis cascade. Small non-coding microRNAs (miRNAs) play a crucial role in the post-transcriptional regulation of gene expression. Mounting evidence highlights the role of miRNAs in malignant hematopoiesis via the regulation of oncogenes and tumor suppressors involved in proliferation, differentiation, and cell death. In this review, we provide current knowledge about dysregulated miRNA expression in the pathogenesis of hematological malignancies. We summarize data about the clinical utility of aberrant miRNA expression profiles in hematologic cancer patients and their associations with diagnosis, prognosis, and the monitoring of treatment response. Moreover, we will discuss the emerging role of miRNAs in hematopoietic stem cell transplantation (HSCT), and severe post-HSCT complications, such as graft-versus-host disease (GvHD). The therapeutical potential of the miRNA-based approach in hemato-oncology will be outlined, including studies with specific antagomiRs, mimetics, and circular RNAs (circRNAs). Since hematologic malignancies represent a full spectrum of disorders with different treatment paradigms and prognoses, the potential use of miRNAs as novel diagnostic and prognostic biomarkers might lead to improvements, resulting in a more accurate diagnosis and better patient outcomes.

## 1. Introduction

Hematologic malignancies represent a set of malignant disorders of the blood and lymphatic systems characterized by rapid progression. Genetic and epigenetic changes result in the clonal proliferation of progenitor and stem cells. Changes in signaling pathways disrupt the self-renewal ability of hematopoietic cells, together with their proliferation and differentiation into other lineages. Due to the high heterogeneity of hematologic cancer subtypes, the evaluations of new diagnostic and prognostic biomarkers are greatly required to improve the outcomes of patients.

Small non-coding microRNAs (miRNAs), first described by Lee et al. [1], represent a group of short regulatory molecules that participate in practically every physiological process, playing a crucial role in the post-transcriptional regulation of gene expression [2,3]. MiRNAs are partially complementary to several mRNAs, and binding to mRNA leads to the degradation or downregulation of gene expression by several mechanisms, including translational repression, mRNA cleavage, and deadenylation [4]. Thus, miRNAs regulate numerous cellular processes, including differentiation, proliferation, migration, and apoptosis. Importantly, miRNAs are critically involved in tumorigenesis and metastatic spreading, and their dysregulation has been described in a wide spectrum of hematologic malignancies [5].

Malignant transformation of hematopoietic cells is associated with changes in the expression levels of oncogenes and tumor suppressor genes. The link between miRNAs and cancer was initially documented in the case of chronic lymphocytic leukemia (CLL) [6]. Altered miRNA biogenesis and post-transcriptional gene silencing have serious consequences for hematopoietic differentiation and proliferation, leading to malignant hematopoiesis. Recently, the results of preclinical and clinical studies have suggested the clinical utility of miRNAs as diagnostic and prognostic biomarkers for treatment response. Mounting evidence has found that miRNA-inducing gene expression changes and heterogenous genetic profiles might serve as potential tools for the personalized treatment of patients receiving hematopoietic stem cell transplantation (HSCT).

Herein, we aim to provide current knowledge about the involvement of miRNAs in the physiology and pathogenesis of hematologic malignancies. The emerging role of miRNAs in HSCT and patient outcomes will be discussed. Moreover, we outline the new research trends and the possibilities of miRNA-based approaches for clinical practice.

## 2. MicroRNA in Hematopoiesis and Hematologic Malignancies

Hematopoiesis is the process of blood cell formation [7,8] in which hematopoietic stem cells (HSCs) generate and form blood cells, including multi-potent progenitors (MPPs) [9]. This continuous process is dependent on the proper expression of transcription factors and consists of several phases [10]. HSCs differentiate into either common myeloid progenitors (CMPs) or common lymphoid progenitors (CLPs) [11]. CMPs later develop into megakaryocytic and myeloid lineages, while CLPs give rise to B- and T-lymphocytes and NK-cells [12,13]. Mounting evidence shows that hematopoietic lineage differentiation is regulated by miRNAs [14,15,16].

### 2.1. MiRNA Biology

MiRNAs represent a group of small non-coding RNA molecules consisting of approximately 22 nucleotide sequences with key regulatory functions. Their post-transcriptional repression of relevant mRNA targets plays a crucial role in the regulation of various biological processes, including cell cycle, differentiation, growth, and cell death [17,18]. Most miRNA sequences are localized in introns or exons of non-coding RNA molecules. However, some miRNAs originate from introns in pre-mRNA (mirtrons). Different miRNA localizations determine canonical or non-canonical pathways [19]. In most cases, miRNA genes are transcribed in the nucleus by RNA polymerase II to primary miRNA (pri-miRNA), followed by cleavage to the precursor (pre-miRNA) structures. Further exporting to the cytoplasm and cleavage by the Dicer complex to small dsRNAs enables their binding to Argonaute proteins (AGO) and the RISC complex, leading to mature single-stranded miRNAs [20]. The canonical mechanism of miRNA biogenesis is dependent on Drosha and DGCR8 proteins, while some non-canonical pathways (e.g., mirtrons) are Drosha- and Dicer-independent processes [21].

Mature miRNAs recognize target mRNAs and mediate mRNA silencing or can be transported from the cell cytoplasm into extracellular circulation. Studies show much higher stability of extracellular miRNAs in comparison to cellular miRNAs [22]. Recently, circulating or cell-free miRNAs analyzed in body fluids represented potential biomarkers of various pathological states, including cancer diagnosis, prognosis, and therapy [23]. Besides their associations with proteins, especially AGO2, extracellular miRNAs can be transported in vesicles, such as exosomes, apoptotic bodies, and microvesicles [24]. Exosomes may also contain mature or pre-miRNA forms since some cancer exosomes contain a protein processing complex (RISC Loading complex) and thus represent cell-independent miRNA maturation [25]. As documented, exosomes function as traffic vehicles to the surrounding microenvironment. They can be received by recipient cells, and subsequent miRNA releasing could mediate changes in gene expression of target genes [26]. Studies concerning the exosomal miRNAs reveal their involvement in cancer pathogenesis with an emerging clinical potential [26].

### 2.2. The Role of microRNAs in Normal Hematopoiesis

The mechanism of miRNA biosynthesis includes a lot of components. During hematopoiesis, loss of Drosha or DGCR in the Dicer-independent mechanism impacts the way miRNAs mature. The absence of AGO2 deactivates the process of miRNA biosynthesis and expression [2,9].

In normal hematopoiesis, miRNAs regulate the differentiation, state, function, and self-renewal ability of HSCs, apoptosis levels, and differentiation of myeloid and lymphoid progenitor cells [27]. Moreover, miRNAs regulate the erythropoiesis, megakaryopoiesis, granulopoiesis, and differentiation of B- and T-lymphocytes [10]. According to the findings, miR-125b regulates the hematopoietic differentiation of HSCs through the modulation of transcription factors for B- and T-cell differentiation via targeting *BLIMP-1, IRF-4, IFNG, IL2RB, IL10RA,* and *PRDM1* [28]. The normal cycling and self-renewal of HSCs are regulated by miR-126 [29] and miR-33 [30], while miR-125b promotes the expansion of HSCs [31].

MiR-155 controls the development of myeloid lineage and differentiation of erythroid progenitor cells [32,33]. Fontana and colleagues found that the monocyte differentiation was regulated by miRNAs 17-5p, 20a, and 106a [34]. Furthermore, miR-223 controlled myeloid differentiation through the LMO, MEF2C, NFI-A, and E2F1 transcription factors [28]. MiR-223 also regulates the differentiation and maturation of granulocytes [35]. Elevated expression promotes the development of mature granulocytes from myeloid progenitors [36]. Basal granulopoiesis is regulated by miR-125a. In addition, miR-125a regulates the production of neutrophils via the G-CSF signaling pathway [37]. Pro-myelocytic cells differentiate into monocytes via cooperation between miR-424 and transcription factors of the myeloid lineage [36].

MiR-146a activates the innate immunity targets involved in megakaryopoiesis [38]. Suppressing the inflammatory cytokines from innate immune cells by miR-146a inhibits the development of megakaryocytes [39]. Overexpression of miR-22 promotes the differentiation of megakaryocyte cells [40]. Zini and colleagues demonstrated that the lineage of granulocytes and megakaryocytes was modulated by the expression of miR-382-5p via downregulation of *MXD1.* Overexpression of miR-382-5p in CD34(+) HSCs resulted in decreased levels of megakaryocyte precursors while granulocytes were elevated [41]. The study of miR-22 functions in hematopoiesis discovered that the miR-22/*MECOM (EVI1)* regulated macrophage differentiation, and that *MECOM* mRNA degradation was mediated by miR-22 [42].

Differentiation of primitive erythroid progenitors from multipotent hematopoietic stem cells drives erythropoiesis. Overexpression of miR-486 multiplies erythroid proliferation and differentiation [43]. The inhibition of miR-150 expression levels enhances terminal erythropoiesis [44]. Kotaki et al. found that in the mouse model, the differentiation of erythrocytes was inhibited by miR-669m overexpression. Using bioinformatic analyses, authors showed that miR-669m targeted the *Akap7* and *Xk* and inhibited erythroid hematopoiesis [45]. The experimental study by Xu et al. demonstrated the impact of miR-451 on the development of erythrocytes. Erythropoiesis was inhibited by miR-144 in CD34+ cells [46]. The mechanisms of the miRNA biogenesis pathway affect erythropoiesis. MiR-451 with Ago2-dependent and Dicer-independent processes regulate the maturation of erythrocytes [47,48].

MiR-128-2 overexpression affects the development of CLPs and immature lymphocytes [14,15]. MiR-24 inhibits B-cell differentiation and induces lymphoid cell apoptosis [49]. Lymphopoiesis of the B-cell lineage is inhibited by the miR-23a cluster [50]. MiR-150 regulates the differentiation of T lymphocytes. During the maturation of the T-cells, miR-150 is upregulated, but during T-cell differentiation into Th1 and Th2 subtypes, miR-150 expression is downregulated [51].

### 2.3. MicroRNA Dysregulation in Malignant Hematopoiesis

The development of hematologic malignancies is linked to chromosomal translocations, mutations, and epigenetic regulation, leading to dysregulation of tumor suppressor or oncogenic pathways. Han et al. reported that alterations in mechanisms of miRNA expression, and thereby in post-transcriptional miRNA regulations, represent critical events in malignant hematopoiesis [52]. Most miRNAs play an oncogenic (oncomiRs) or tumor-suppressive role, but in some cases, miRNAs might have a dual function [5].

According to the findings, abnormal miRNA expression is caused by chromosomal aberration, epigenetic deregulation of miRNA, and alterations in miRNA biosynthesis [53,54]. In addition, DNA methylation of tumor suppressor miRNAs and histone modification represent epigenetic mechanisms, which also contribute to malignant hematopoiesis [55]. Aggire et al. found that miR-124a expression was associated with decreased levels of 3mK4H3 and elevated 2mK9H3 markers, which promote the closed chromatin structure [56].

An aberrant miR-126 transcription plays a role in the leukemogenesis of acute myeloid leukemia (AML). An increased miR-126 expression was documented in a subpopulation of leukemic stem-like cells, which correlated with poor survival and a higher chance of relapse in AML patients [57]. As shown, miR-3662 reduced the growth and survival of AML cells in vitro. Interaction of miR-3632 and *HBS1L–MYB* regions located at the 6q23.3 chromosome affected hematopoiesis, especially erythroid differentiation, and leukemogenesis [58]. Lu et al. documented a dramatic upregulation of miRNA-301b-3p related to increased proliferation and inhibited apoptosis in AML cells [59]. In chronic myelogenous leukemia (CML), leukemogenesis is promoted by miR-17-92 targeting the *A20* gene via the NF-kB signaling pathway. Jia et al. described the mechanism of aberrant leukemogenesis by miR-17-92 in the mouse model of CML, showing that overexpressed *A20* induced apoptosis and inhibited cell proliferation [60]. The study of the long non-coding RNA (lncRNA) *MALAT1*/miR-328 axis provided data that CML development was suppressed after miR-328-dependent *MALAT1* knockdown inhibited proliferation and led to cell cycle arrest [61]. Furthermore, miR-146a displays antitumor activity in myeloid leukemia via regulation of the NF-κB pathway by targeting *IRAK1* and *TRAF6* transcripts. C-miR-146a, a miR-146a mimic oligonucleotide conjugated to a scavenger receptor/Toll-like receptor 9 agonist reduces the expression of miR-146a targets and blocks NF-κB activation [62]. The correlation between aberrant miRNA expression and reduced apoptosis was observed in several hematologic malignancies [5,63]. In the human megakaryoblastic leukemia cell line MEG-01, derived from a patient with CML, expression of miR-15a and miR-16-1 promoted apoptosis by repression of the anti-apoptotic *BCL-2* gene [64]. The results from in vitro and in vivo analyses showed that miR-125b inhibited the apoptosis in hematopoietic cells via repressing the *Trp53inp1* gene [65].

Several studies focused on the variable expression of miRNA in myeloproliferative neoplasms. A comparison of miRNA expression levels in patients and healthy controls showed upregulated levels of miR-125b-5p and miR-125a-5p in polycythemia vera (PV) as well as in essential thrombocythemia (ET) patients. These findings highlight the possible role of aberrant miR-125 expression in the phenotype of patients with myeloproliferative malignancies [66]. Bruchova et al. investigated the relationship between dysregulated miRNAs and PV pathophysiology. The results documented the downregulation of let-7a and upregulation of miR-182 in granulocytes of PV patients. Furthermore, upregulation of miR-143, miR-145, miR-223, and high miR-26b levels were found in PV mononuclear cells and platelets, respectively. On the other hand, reticulocytes from PV patients showed miR-30b, miR-30c, and miR-150 downregulation [67]. Gebauer et al. aimed to identify potential miRNA biomarkers for differentiation between the subtypes of myeloproliferative neoplasia, including PV, ET, and early primary myelofibrosis (PMF). According to the findings, alterations in the expression of miR-10a and miR-150 were demonstrated for ET and PMF and also for PV and PMF, respectively. Moreover, the authors observed a correlation between miR-150 with a *JAK2* allele burden and peripheral blood counts [68].

Kuang et al. studied the impact of miR-378 overexpression on myelodysplastic syndrome (MDS), showing that elevated levels of miR-378 promoted apoptosis and inhibited the growth of leukemic cells by activating the intrinsic and extrinsic pathways [69].

MiR-21 and miR-221/222 are oncogenes allowing increased cell proliferation and growth in multiple myeloma (MM) [70,71], while miR-342-3p suppresses MM development [72]. Long et al. revealed that miR-140-3p acts as a tumor suppressor by decreasing the proliferation and inducing the apoptosis of MM cells [73].

In T-cell acute lymphoblastic leukemia (T-ALL), DNA methylation in the CpG promoter region leads to miR-204 downregulation, while miR-204 overexpression in vitro inhibits the proliferation and enhances the apoptosis of T-ALL cells [74]. In CLL, reduced expression of pro-apoptotic miR-15a/16-1 is critical for cell survival. Kasar et al. reported the relationship between miR-15a/16 overexpression, followed by increased apoptosis of malignant cells and inhibition of histone deacetylation and the knockdown of B-cell-specific activator protein [75]. Recently, decreased levels of miR-125a and miR-223, together with an increased expression of their targets *BCL-2* and *STAT3*, were identified in CLL patients [76].

Tumor suppressor miR-34a was found methylated in 75% of lymphoma cell lines [77]. MiR-155 regulates histone deacetylase 4 and transcriptional machinery in B-cell lymphoma 6 (BCL6), leading to leukemogenesis [78]. In aggressive B-cell lymphomas, miR-29 suppresses tumorigenesis through the knockdown of histone acetylation [79]. Meyer et al. found the inhibition of *TAL1* expression by miR-17-92 cluster in primary human CD34+ cells. Deregulation in hematopoietic stem cells, caused by the TAL1 transcriptional complex, impaired erythroid differentiation. At the erythroid lineage, differentiation and proliferation of HSCs are modulated by the miR-17-92/*TAL1* complex and can promote lymphoma development [80]. Zhou et al. found that cell proliferation and apoptosis of mantle cell lymphoma were suppressed by increased expression of miR-223 [81].

More details about miRNAs and their targets participating in hematologic malignancies are summarized in Table 1.

### 2.4. Aberrant microRNA Expressions in Diagnosis, Prognosis, and Monitoring of Therapy

Due to the origin of hematologic malignancies in the bone marrow and lymph nodes, miRNA expression analyses were performed in peripheral blood, plasma and serum, and bone marrow aspirates. MiRNA passes into circulation directly via apoptotic or necrotic events, or they are actively secreted by both normal and cancer cells. MiRNAs become highly stable when they are encapsulated in microvesicles or exosomes. This allows better miRNA transport and mediates the cell–cell communication between cancer cells, as well as between cancer cells and surrounding non-malignant cells [88,89].

Similarly to other cancers, an increasing number of exosomes was found in hematologic malignancies compared to normal blood samples. They extensively influence numerous pathological processes, including tumor survival, progression, and therapy resistance [90,91]. Moreover, exosomal miRNAs could possess a smaller amount of unspecific background compared to free extracellular miRNAs [92]. Several studies concerning hematologic malignancies assessed the correlations between exosomal miRNAs and diagnosis, prognosis, and chemotherapy resistance [93,94,95,96,97,98]. The results of aberrant miRNA expressions in acute and chronic leukemias, lymphomas, and myelomas, whose associations were evaluated with regard to any clinical consequences, are summarized in Table 2.

The majority of presented miRNA studies were conducted in pediatric patients with a diagnosis of the most common pediatric malignancy, ALL, in BM aspirate samples [100,101,102,103,104,105,106,107,108]. The researchers identified specific miRNAs for discrimination between several genetic, and T-ALL and B-ALL subtypes [104,105]. Other up- and downregulated miRNAs are associated with the prediction of early relapse [107] and drug resistance [102,103,104]. In adults with acute leukemias, upregulated miR-128a and miR-128b together with downregulated let-7b and miR-223 discriminated ALL from the most common AML in adult patients [109]. Furthermore, miR-363 seems to be a prognostic factor for optimal therapeutic strategy in AML. In patients with higher miR-363 expression in PB samples, early allogeneic transplantation (allo-HSCT) was recommended as a more effective strategy compared to chemotherapy but improved OS was found in those with a low level of miR-363 [110]. Several adult CML studies identified a single or set of specific miRNAs, which could be useful for the prediction of imatinib response [120,121,123,125]. In MM patients, the aberrant expression of other miRNA was found mostly in association with shorter PFS and OS [95,127,129,130,131,133,134,135,137] and bortezomib response [94,127]. In patients with leukemias and lymphomas, the opposite tendency in the expression of specific miRNAs was detected after the chemotherapy compared to the state before treatment. These findings might be used for therapy management.

Among the summarized results, most were performed in malignant samples. Only 30% of studies investigated miRNA expressions in plasma or serum samples, where the extracellular miRNA molecules originating from both cancer and normal cells coexist. It is generally assumed that exosomes are secreted mainly by live cells, which influence many physiological and pathological processes. Thereby, exosomal miRNA from patients with hematologic malignancies could be less contaminated by the background from the apoptotic and necrotic cells [89]. Moreover, several panels of exosomal miRNA were identified for early detection, discrimination of different subtypes, and prediction of drug resistance [89,93,94].

## 3. MicroRNA in Hematopoietic Stem Cell Transplantation

### 3.1. Hematopoietic Stem Cell Transplantation

Hematopoietic stem cell transplantation (HSCT) is a routine procedure for treating malignant and non-malignant diseases [138]. The pre-transplant course, known as the conditioning regimen, consists of chemo- or radiotherapy, or their combination, aiming to eradicate cancer cells and prepare recipient bone marrow for new donor cells [139]. Transfer of healthy HSCs helps to augment the function of the recipient bone marrow to generate functional cells [140]. For autologous transplantation (auto-HSCT), stem cells are obtained from the recipient before conditioning, cryopreserved, and then reinfused. On the other hand, allo-HSCT uses stem cells obtained from related/unrelated donors. Donors of HSCs might be family members, volunteers, or banked umbilical cord blood cells [141]. Human leukocyte antigen (HLA) matching between donors and recipients plays a key role in transplantation outcomes [142]. Over the past twenty years, data has documented increasing transplant rates for auto/allo- HSCT [143].

In the case of cord blood as a source of HSC, HLA matching requirements are less stringent due to the naive immunity of newborns. The risk of a rejected graft is less in the case of mismatched cord blood cells than transplantation of adult peripheral blood cells [144,145]. One of the main concerns of allo-HSCT is a high incidence of graft-versus-host disease (GvHD). GvHD is associated with the immunological attack and rejection of host tissue by donor alloreactive T lymphocytes [146]. However, in the case of identical twins as donor and recipient, there is no risk of GvHD and no failure of graft in the recipient [140].

Acute and chronic GvHD represent major specific post-transplant complications affecting patient life. The differences between these two forms are the time of onset, clinical features, and immunopathological mechanisms [147]. An acute GvHD (aGvHD) develops within the first 100 days post-transplantation and primarily affects the gastrointestinal tract, liver, skin, eyes, and oral mucosa [148]. A chronic GvHD (cGVHD) represents a delayed complication associated with transplantation, significantly reducing the quality of life [149]. The onset of cGvHD is more than 100 days post-transplantation, and the incidence varies from 30 to 70% of patients [150,151]. An elevated occurrence of cGvHD is documented in pediatric patients [152]. Transplant-caused mortality and both aGvHD and cGvHD were reduced in the case of T-cell depletion using antibodies. Results showed that T-cell depletion might prevent GvHD only in patients with early-stage leukemia and low relapse risk [153]. Timely diagnosis of GvHD is very important for patient outcomes, and early biomarkers for the prevention of morbid complications associated with HSCT need to be uncovered [154]. Recently, immune activation markers, organ-specific markers, and miRNAs as systemic markers are widely studied in routine use for GvHD prognosis and diagnosis [155,156].

### 3.2. MicroRNA and Graft-Versus-Host Disease

**Figure 1:**
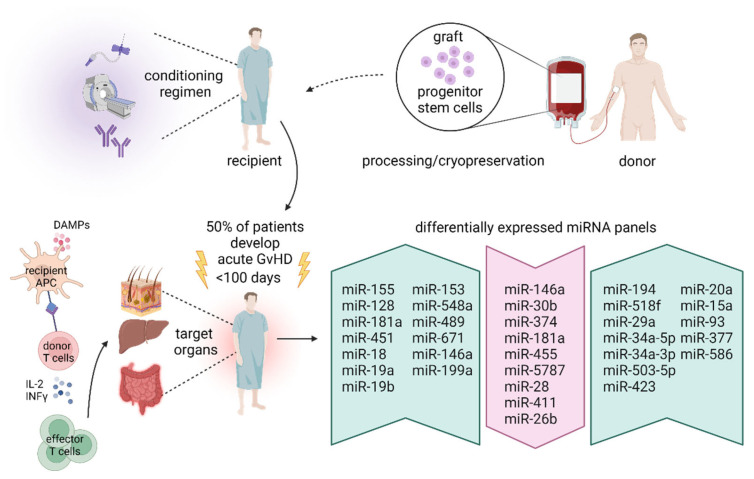
MiRNAs as potential diagnostic and prognostic biomarkers for aGvHD in patients receiving HSCT. The onset of aGvHD limits the successful therapy of hematologic malignancies. Conditioning regimens damage the host tissue with the induction of dangerous signals, including damage-associated molecular patterns (DAMPs). DAMPs lead to the activation of antigen-presenting cells (APCs), which subsequently stimulate the proliferation of donor T cells into effector T cells. When entering the target organs, effector T cells cause aGvHD development. Aberrant miRNA expression is considered as being involved in the pathology and onset of aGvHD. Selected miRNAs might be used as noninvasive diagnostic biomarkers due to their differential expression in plasma samples of patients with aGvHD compared to patients without GvHD. The panels on the right side of the figure represent upregulated (green arrows) and downregulated (red arrow) miRNAs in HSCT patients who developed aGvHD. Abbreviation: aGvHD, acute graft-versus-host disease; APCs, antigen-presenting cells; DAMPs, damage-associated molecular patterns; HSCT, hematopoietic stem cell transplantation.

Several preclinical and clinical studies in various periods pre- and post-HSCT showed dysregulated miRNA levels and their function in massive immune responses leading to the development of GvHD [157,158]. A differently expressed miRNA profile appears to be a promising noninvasive tool for specific diagnosis and prediction of aGvHD occurrence (Figure 1).

MiR-146a and miR-155 are among the most studied miRNAs in association with GvHD. MiR-155 plays an important role in T-cell proliferation, affecting the T and B lymphocyte function. Significantly upregulated miR-155 was observed in the serum of hematologic cancer patients with aGvHD compared to non-aGvHD patients. Moreover, a higher miR-155 expression correlated with higher grade (III-IV) aGvHD patients and elevated levels of soluble IL-17, IL-9, and IFN-gamma factors. These are specific for donor T-cell activation and tissue damage during aGvHD [159]. Importantly, upregulated miR-155 was found also in endothelial microparticles from peripheral blood. The frequent presence of microparticles is common for GvHD, and the increasing peak of miR-155 levels in these particles occurred even before miR-155 elevation in T-lymphocytes. In addition, miR-155 inhibition induced aberrant Th and Treg cell differentiation. Delivery of miR-155 by endothelial microparticles is involved in specific functions of T-lymphocytes and could mediate the onset of aGvHD [160]. MiR-146a is a key controller of immune T-cell response. In a mouse model, the overexpression of miR-146a was associated with reduced GvHD severity caused by the negative regulation of T-cells from donors by targeting TNF receptor-associated factor 6 (TRAF6), and subsequent TNF transcription inhibition [161]. Moreover, authors observed that SNP polymorphism rs2910164 in the precursor miR-146a of HSCT donors and recipients predisposes them to a higher risk for severe aGvHD [161,162]. According to the findings, the downregulation of miR-146a in the 28-day period after allo-HSCT correlated with a higher incidence of aGvHD. Furthermore, the statistical model concerning miR-146a and miR-155 expression in the peripheral blood showed a significant biological synergy [163].

For a complex view, Crossland et al. analyzed miR-146a and miR-155 levels in skin and gastrointestinal biopsies and various types of body fluids, including serum and urine. In all sample types, higher levels of both miRNAs were detected in aGvHD patients compared to non-GvHD patients. In addition, the upregulation of miR-146a and miR-155 in the 14-day period after HSCT was found in serum samples of patients who later developed aGvHD. Nevertheless, expression results of both miR-146a and miR-155 supported their potential to be biomarkers for GvHD development [164].

In relationship to aGvHD, many authors found the upregulation of miR-423, miR-199a, miR-93, and miR-377 and detected an association between their higher expression and diagnostic or prognostic ability and GvHD severity [165,166]. A broad-range miRNA profiling study analyzing 799 mature miRNAs in serum samples detected 61 miRNAs with different expressions in aGvHD. Among them, 10 miRNAs were re-analyzed in another patient cohort. Significant upregulation of miR-20a and miR-15a and downregulation of miR146a, miR-30b, miR-374-5p, and miR-181a were verified in aGvHD patients. However, receiver operating characteristic (ROC) analysis suggested diagnostic potential only for miR-30b, miR-374-5p, and miR-15a expression. On the other hand, prognostic significance demonstrated upregulation of miR-18, miR-19a, miR-19b, miR-20a, miR-146a, and miR-451 in a 14-day post-HSCT period before GvHD onset [167]. Xie et al. found contradictory results, showing higher miR-181a expression in aGvHD compared to non-aGvHD patients. Moreover, the results found a correlation between the severity of GvHD and a positive or negative correlation with serum IL-2, IL-17a, IL-22, and IL-13 cytokines. Further investigations are needed to elucidate the role of miR-181a as a promising GvHD biomarker [168].

Upregulation of miR-194, miR-518f, miR-29a, miR-586, miR-153, and miR-548a and downregulation of miR-455 and miR-5787 in plasma samples significantly mediated aGvHD development in post-HSCT patients [169,170,171,172,173]. Zhang et al. evaluated a diagnostic value of novel serum miRNAs in aGvHD, including lower miR-28 and higher miR-489 and miR-671 expression levels. A predictive model for increased GvHD risk identified two downregulated miR-26b and miR-374a. In addition, the results uncovered the role of miR-411 in aGvHD monitoring since the decreased expression of miR-411 was detected in the case of aGvHD development, compared to higher miR-411 levels when aGvHD was under control [174]. Exosomal miRNA expression represents a promising diagnostic biomarker for late-onset aGvHD. Using a low-density miRNA array, differences in expression profiles of 55 miRNAs were identified between late-onset aGvHD and non-aGvHD. Higher expression of miR-128 was validated for diagnosis of late-onset aGvHD. As known, miR-128 target genes play a role in inflammation and immune response pathways [175].

The changes in miRNA expression profiles were also detected in cutaneous aGvHD. Differentially expressed let-7c, miR-503, miR-365a, miR-34a-5p, and miR-34a-3p were shown in pre-HSCT, post-HSCT, and control skin biopsies [176].

Studies concerning diagnostic and prognostic biomarkers for cGvHD are still missing [177]. Recent results identified the distinction between miRNAs in cGvHD patients. The regression model and bioinformatic analysis showed that the upregulation of miR-365a, miR-148a, and miR-378a in plasma samples had diagnostic relevance for patients one year post-HSCT with cGvHD symptoms [178]. In plasma extracellular vesicles (EVs), Lacina et al. detected differentially expressed miRNA characteristics for cGvHD. Upregulation of miR-29c and downregulation of miR-630 and miR-374b were found in cGvHD compared to non-cGvHD patients [179].

### 3.3. MicroRNA and Post-HSCT Patient Outcomes

MiRNA expression profiling suggests that numerous miRNAs are associated with mortality risk in HSCT patients. The group of high-risk patients with a pre-transplant comorbidity index score ≥4 compared to low-risk patients with index score 0 showed significantly downregulated miR-374b and miR-454 but upregulated miR-142, miR-191, miR-424, miR-590, miR-15b, and miR-29c. These results outline the remarkable predictive potential of miRNA expression for patients’ outcomes [180]. Cheng et al. noticed that AML patients who underwent allo-HSCT and showed high miR-99a expression had poor outcomes. MiR-99a upregulation represented an unfavorable prognostic marker for EFS and OS in multivariate analysis [181]. In addition, the miRNA expression profile can stratify patients who profited from allo-HSCT or chemotherapy. A better OS and EFS were detected in the AML group receiving allo-HSCT and expressing low levels of miR-425 compared to patients undergoing chemotherapy [182]. Furthermore, miR-363 could serve as a prognostic marker for an appropriate therapeutic strategy with improved clinical outcomes in the allo-HSCT group, since higher miR-363 expression was associated with improved OS and EFS [110].

Survival analyses were included in several clinical studies concerning the relationship between miRNA expressions and GvHD. Four-miRNA panels consisting of miR-423, miR-199a-3p, miR-93, and miR-377 were analyzed in plasma samples and showed that high-risk patients with upregulated miRNAs had a poorer outcome than low-risk patients. Moreover, the elevated expression of miRNA panels appeared as an independent prognostic factor for shorter OS in aGvHD patients [165]. In contrast, higher expression of miR-19b, miR-20a, and miR-30b at the time of diagnosis was associated with improved OS [167].

The association between the miRNA profile and cutaneous aGvHD was investigated in skin samples, showing that underexpressions of miR-503 and miR-34a-3p were implicated in improved OS in post-HSCT patients. The results documented overexpression of miR-503, mir-34a-5p, and miR-34a-3p in the serum of aGvHD patients, suggesting their potential use as circulatory biomarkers for cutaneous aGvHD development [176].

Lim et al. investigated the association between miRNA expression from EVs and clinical complications of allo-HSCT patients. Several correlations with infectious complications were observed for five different EV miRNAs, miR-223-3p, miR-21-5p, miR-23a-3p, miR-375, miR-423-5p, for positive, and miR-425-5p, miR-342-3p, miR-320b, miR-454-3p, miR-151a-3p for negative correlations, respectively [183]. Allo-HSCT with myeloablative conditioning is frequently accompanied by post-transplantation complications, including gastrointestinal toxicity, mucositis, and overall inflammation. Upregulation of miR-155 and downregulation of miR-146a are significantly associated with markers of gastrointestinal toxicity, with IL-6 and CRP representing plasma inflammatory factors. According to the findings, these miRNAs could play a critical role in inflammation response [184].

MiRNA expression was shown to influence engraftment efficacy. Higher expression of miRNA-15a, miRNA-16, miRNA-126, and miRNA-146a before auto-HSCT and early post-HSCT were positively correlated with a longer period until engraftment in patients with MM and lymphoma [185]. Rafiee et al. observed a specific correlation between the upregulation of miR-155 and platelet and neutrophile engraftment, suggesting miR-155 as a potential predictor of auto-HSCT patient outcome [186]. Similarly, higher expression of miR-193a-5p in MM patients before auto-HSCT predicted a reduction in early relapse and better PFS [187].

## 4. Investigating the Therapeutical Potential of the miRNA-Based Approach in Hemato-Oncology

Higher expression of oncogenic miRNA might be regulated by several strategies [52]. AntagomiRs represent antisense oligonucleotides prepared by genetic engineering, which are capable of miRNA silencing [188]. Dorrance et al. suggested the potential therapeutic option of antisense miRNA in AML patients, showing that antagomiR-126 reduced the number of leukemia stem cells in vivo [189]. According to these findings, treatment with imatinib led to the upregulation of miR-21 in Philadelphia chromosome-positive ALL cells. The application of antagomiR-21 demonstrated positive results in sensitizing cells to imatinib, inducing cancer cell apoptosis, and inhibiting the phosphatidylinositol-3-OH kinase/AKT (PI3K/AKT) pathway [190]. *CBFB-MYH11* (CM) gene fusion upregulated the levels of miR-126 in AML, which led to the survival of leukemia-initiating cells. As shown, the genetic deletion of miR-126 prolonged survival in a murine model with CM gene fusion. Treatment with therapeutic anti-miR-126 in combination with chemotherapy effectively inhibited AML cell progression [191]. The positive effect of antagomiR-196b and antagomiR-21 was documented in the MLL-AF9–initiated leukemia-murine model with no changes in murine behavior or vital organ functions [192]. The results from an in vitro study on diffuse large B-cell lymphoma (DLBCL) cell lines revealed an attenuated proliferation and increased apoptosis after the application of an oligonucleotide miR-155 inhibitor. In addition, the delivery of the miR-155 inhibitor into a DLBCL-bearing murine model led to a decrease in tumor volume [193].

Accumulating evidence suggests that circular RNAs (circRNAs) also play a key role in hematopoiesis and hematologic malignancy initiation [194] and could be exploited in therapy. CircRNAs represent non-coding RNAs that contain a covalently closed loop structure. In the vast majority of eukaryotes, the loop originates mainly from the reverse splicing of pre-mRNA transcripts [195]. CircRNAs have multiple binding sites for miRNAs and, among other functions, could act as endogenous miRNA sponges by sequestering and preventing them from interacting with their target mRNAs [196]. Recent studies have pointed out the possibilities of using circRNA in the inhibition of miRNAs, which are related to cell proliferation, apoptosis, invasion, and migration of cells in hematologic malignancies. Han et al. revealed the ability of circ-0001947 to suppress the proliferation of AML cells by inhibiting miR-329-5p [197]. The expression of circ-KEL, in turn, can inhibit apoptosis of AML via sponging miR-335-5p [198], and circ-0009910 was associated with inhibition of apoptosis by sponging miR-34a-5p and miR-5195-3p in CML and AML, respectively [199,200]. In addition, circ-0069767 was found to reduce the invasive and migratory capacities of MM cells by sponging miR-636 with an impact on the regulation of *K-RAS* expression [201].

On the other hand, numerous miRNAs were downregulated in hematologic malignancies, leading to worse treatment outcomes. A significantly lower level of miR-29b was detected in blood mononuclear cells from patients with AML or CML, which correlated with poor treatment-free survival [202,203]. Garzon et al. documented the tumor suppressor effect of synthetic miR-29b oligonucleotides as a potential therapy option for AML. Synthetic miR-29b directly injected into murine tumors reduced tumor growth. Results from in vitro experiments showed the association between transfection of synthetic miR-29b and downregulation of anti-apoptotic *MCL-1*, leading to reduced cell growth [83]. Furthermore, synthetic miR-29b oligonucleotides effectively downregulated the expression of *DNMT1, DNMT3A*, and *DNMT3B* in AML cells. Prepared hypomethylating compound miR-29b increased promoter hypomethylation and reactivated the expression of *p15INK4b* and *ESR1* genes [204]. A previous study by Blum et al. showed that the reexpression of both genes was associated with clinical response to therapy in leukemic patients treated with decitabine [205].

A higher expression of the *ST8SIA4* gene correlates with leukemia multidrug resistance via the elevated activity of the PI3K/AKT pathway. In vivo experiments confirmed that the upregulation of miR-181c in combination with adriamycin significantly decreased tumor growth in adriamycin-resistant mice. Decreased level of *ST8SIA4* was observed in tumors injected with miR-181c. Based on this result, inhibition of the PI3K/AKT pathway helped to target sensitive cancer cells in treatment and suppressed drug resistance in a murine model [206].

Nevertheless, free synthetic miRNA might be degraded in biofluids, and its cellular uptake is limited. The nanoparticle delivery system represents a novel strategy for miRNA delivery. Transferrin-conjugated nanoparticle delivery for synthetic miR-29b (Tf-NP-miR-29b) led to a 200-fold increase in mature miR-29b. In a leukemic murine model, treatment with Tf-NP-miR-29b led to more prolonged survival than in mice treated with free miR-29b. Moreover, other miR-29b targets were decreased, including *KIT, DNMT3A, DNMT3B, DNMT1, SP1, CDK6*, and *FLT3* [207]. In another study, Huang et al. used lipopolyplex nanoparticles for more efficient delivery of miR-181a into KG1a, MV4-11, and OCI-AML cells. In a leukemic murine model, the administration of Tf-NP-miR-181a caused an increased level of miR-181a, reduced tumor burden, and prolonged mice survival [208]. Since higher expression of miR-181a in patients with AML correlated with longer survival and full remission compared to patients with low miR-181a levels [209], increasing miR-181a might bring a potential clinical benefit.

## 5. Conclusions and Future Directions

Due to the aggressive nature of hematologic malignancies, early and accurate diagnosis is the key to improving prognosis, treatment results, and patient survival. The discovery of miRNAs triggered intensive research aiming to determine their role in normal and malignant hematopoiesis. Altered miRNA levels in tumor and healthy cells enable the potential use of circulating miRNAs as diagnostic biomarkers. Mounting evidence describes the role of miRNAs in post-transplant complications, including mainly GvHD, which is the primary cause of premature mortality in patients receiving HSCT. Early detection of aGvHD is critical for prolonging patients’ OS. Since diagnostic and prognostic biomarkers for GvHD have not been determined yet, this research area represents a crucial experimental and medical challenge. Circulating miRNA levels in plasma or EVs represent a promising tool not only for aGvHD but also for cGvHD diagnosis, prognosis, and prediction.

Recently, the identification of predictive exosomal miRNA panels for drug resistance opens up possibilities for potential intervention. In this context, activation or inhibition of deregulated miRNAs appears to be an attractive therapeutic option for improving treatment efficacy. Animal models and in vitro findings evaluated the clinical significance of reducing oncogenic miRNAs by synthetic antagomiRs and restoring critically reduced levels of tumor-suppressor miRNAs through mimetics. A proper understanding of existing correlations is needed prior to routine clinical application. The research is particularly complicated in the case of dual miRNAs, showing both oncogenic and tumor suppressor activity. Safety and an effective way of delivery represent the major concerns.

Although miRNA’s association with HSCT, post-transplant complications, and patient outcome is a relatively new research area, some results have suggested miRNAs as potentially relevant prognostic and predictive markers. However, further investigations of miRNA dysregulation in hematopoiesis may bring novel strategies in miRNA-based approaches to improve outcomes for hematologic cancer patients.

## Figures and Tables

**Table 1:** MicroRNA expression associated with malignant hematopoiesis.

Malignancy	miRNA	miRNA Expression Level	Target Genes	Study Type	miRNA Function/Clinical Consequence	Reference
AML	miR-3662	DOWN	*IKBKB*	AM, PS (N = 20), TC	Acceleration of the growth and colony formation of HP cells, survival of leukemic cells	[58]
	miR-9	UP	*RHOH* *RYBP + 15 other potential targets*	AM CL PS (N = 85)	Increasing cell survival and decreasing apoptosis	[82]
	miR-29b	DOWN	*MCL-1*, *CXXC6*, *CDK6*	AM, CL, PS (N = 100)	Cell growth and anti-apoptotic activity	[83]
	miR-126	UP	*SLC9A7, ABCG1, MEF2C, RBMPS, LYZ, CSTA, HAL*	AM, CL, PS (N = 6)	Cell growth and anti-apoptotic activity	[57]
	miR-301b	UP	*FOXF2*	CL	Cell proliferation and decreasing apoptosis	[59]
CML	miR-19b (miR-17-92 cluster)	UP	*A20*	AM, BM, CL	Cell proliferation, cell cycle, and decreasing apoptosis	[60]
	miR-328	DOWN	*PIM-1,* *TCF-4*	CL	Cell proliferation, survival	[61]
MDS	miR-378	DOWN	*CDC40*	CL, PS (BM) (N = 20)	Inducing the apoptosis and blocking the cell cycle of MDS cells	[69]
MM	miR-21	UP	*PTEN, Rho-B, BTG2*	AM, CL	Growth and anti-apoptotic activity	[70]
	miR-221/222	UP	*p27Kip1, PUMA, PTEN, p57Kip2*	AM, CL	Proliferation and cell survival	[71]
	miR-342-3p	DOWN	*FOXQ1* *RAP2B* *CDC42*	CL, PS diagnostic and relapsed MM (N = 93)	Methylation-derived silencing of miR-342-3p might be an early event in MM pathogenesis	[72]
	miR-140-3p	DOWN	*BZW2*	AM, CL	Cell proliferation, decreasing the apoptosis in MM cells	[73]
	miR-125a-5p	UP	*TP53*	CL	Cell proliferation, cell growth decreasing apoptosis of cancer cells	[84]
	miR-9	DOWN	*FGFR1* *CDK6*	CL PS (N = 200)	MiR-9 hypermethylation lead to the activation of oncogenic pathways and represents a prognostic factor for survival	[85]
ALL	miR-124a	DOWN	*CDK6*	AM, CL, PS (N = 353)	Cell proliferation and growth of ALL cells	[56]
T-ALL	miR-204	DOWN	*IRAK1,* *NF-kB*	AM, CL, PS (N = 32)	T-ALL growth and metastasis by increased *IRAK1* and activation of *NF-kB* signaling pathway and targets	[74]
CLL	miR-125a, miR-223	DOWN	*BCL2,* *STAT3*	PS (N = 30)	Control of white blood cell production	[76]
	miR-22	UP	*PTEN*	PS (N = 22)	B-CLL cell proliferation	[86]
	miR-15a, miR-16-1	DOWN	*DLEU2*	AM, PBMC	Decreasing apoptosis and cell cycle of malignant B-cells	[75]
NHL	miR-34a	DOWN	*TP53,* *CDK6*	CL, PS (N = 32)	MiR-34a is preferentially hypermethylated in NHL, the role of miR-34a in lymphomagenesis	[87]
	miR-29	DOWN	*IGF-1R, CDK6*	CL	Cell survival and growth regulation in MCLs	[79]
DLBCL	miR-155	UP	*HDAC4*	AM, CL	Block the development of B cells at the immature stage and induce cell proliferation	[78]

Abbreviations: ALL, Acute lymphocytic leukemia; AM, animal model; AML, acute myeloid leukemia; BM, bone marrow samples; CL, cell lines; CLL, chronic lymphocytic leukemia; CML, chronic myelogenous leukemia; DLBCL, diffuse large B-cell lymphoma; MCLs, mantle cell lymphomas; MDS, myelodysplastic syndrome; MM, multiple myeloma; N, number of patients; NHL, Non-Hodgkin’s lymphoma; PBMC, peripheral blood mononuclear cells; T-ALL, T-cell ALL; TC, tissue culture; PS, patient study; T-cell acute lymphoblastic leukemia; UP/DOWN, up-/downregulation of miRNA expression.

**Table 2:** Aberrant miRNA expression in patients with different types of hematologic malignancies with potential for clinical utility in the diagnosis, prognosis, and monitoring of therapy response.

Malignancy	MiRNA	Sample Type	Expression Pattern	No. of Patients	Diagnosis	Prognosis	Therapy Response	Ref.
ALL	miR-146a	Plasma	UP	N = 66	Diagnostic marker pediatric and adult ALL		DOWN after CHT	[99]
ALL pediatric	miR-155a	BM	UP	N = 45		MRD, poor prognosis	DOWN after CHT	[100]
	miR-155	BM	UP	N = 42		Poor outcome		[101]
	miR-200c and miR-326	BM	DOWN	N = 46			Drug resistance	[102]
	miR-324-3p and miR-508-5p	BM	DOWN	N = 50			Drug resistance	[103]
	let-7b, miR-511, and miR-708 miR-196a, miR-383, and miR-542-5p	BM, PB	DOWN UP	N = 81	Genetic subtype discrimination			[104]
	miR-99a, miR-100, and miR-125b		UP				Drug resistance	
	miR-10a, miR-33, miR-134, miR -214, miR -215, miR-369-5p, miR -484, miR-496, miR-518d, miR-572, miR-580, miR-599, miR-624, and miR-627		UP or DOWN			Clinical outcome		
	miR-151a-5p, miR-151b, miR-195-5p, miR-371b-5p, miR-425-5p, miR-455-5p, miR-497-5p, miR-574-5p, miR-708-5p, and miR-1266-5p	BM, PB	DOWN	N = 16	T-ALL and B-ALL discrimination			[105]
	miR-29c-5p, miR-424-5p, miR-450a-5p miR-450b-5p, miR-542-5p, and miR-629-5p		UP					
B-ALL pediatric	miR-21	BM, PB	UP	N = 75		Shorter DFS and OS		[106]
	miR-101-3p, miR-631, miR-922, miR-1324, miR-4699-5p, and miR-4774-5p	BM	UP	N = 40		Prediction of early relapse		[107]
Precursor B-ALL pediatric	miR-151-5p and mR-451	BM	DOWN	N = 189		Shorter RFS		[108]
	miR-1290		UP					
ALL and AML	miR-128a and miR-128b	BM	UP in ALL	N = 136	ALL and AML discrimination			[109]
	let-7b and miR-223		DOWN in ALL					
AML	miR-125b	Exosomes	UP	N = 154		Higher risk of relapse and overall death		[97]
	miR-363	PB	UP	N = 162		Shorter EFS and OS	Preference allo-HCST to CHT	[110]
	miR-504-3p	Serum	DOWN	N = 134		Shorter OS		[111]
	miR-199b-5p, miR-301b, miR-326, miR-361-5p, miR-625, and miR-655	Plasma	UP	N = 8			DOWN after CHT	[112]
AML pediatric	miR-183	BM, serum	UP	N = 106		Shorter PFS and OS		[113]
	miR-199a	BM	DOWN	N = 71	Higher BM blasts	Shorter EFS	Lower therapy response	[114]
	miR-370	BM, serum	DOWN	N = 106		Shorter RFS and OS		[115]
	miR-146a, miR-509, miR-542, and miR-3667	unknown	UP	N = 229 *		Shorter OS		[116]
CLL	miR-32-5p, miR-98-5p, and miR-374b-5p	PB	DOWN	N = 32	Early diagnosis			[117]
B-CLL	miR-145-5p and miR-185-5p	Plasma	DOWN	N = 40	B-CLL detection			[118]
CML	miR-142-5p	BM, PB	DOWN	N = 45			Drug resistance	[119]
	miR-146a	Plasma	DOWN	N = 60			Prediction of imatinib response	[120]
	miR-150	Plasma	DOWN	N = 60			Prediction of imatinib response, UP after CHT	[121]
	miR-150	PB	DOWN	N = 24		Potential marker for blast crisis and hematologic relapses		[122]
	miR-486-5p	PB	DOWN	N = 36	Early diagnosis		Prediction of imatinib response, UP after CHT	[123]
	miR-20, miR-106, and miR-222,	Plasma	UP	N = 50			Potential markers for therapy response	[124]
	miR-122 and miR-126	PB	DOWN	N = 100			Prediction of imatinib response, UP after CHT	[125]
DLBCL	miR-99a-5p and miR-125b-5p	Exosomes	UP			Shorter PFS	CHT resistance	[98]
	miR-199a and miR-497	LN biopsies	UP	N = 63		Longer OS	Drug sensitivity DOWN after CHT	[126]
MM	miR-19a	Serum	DOWN	N = 108		Shorter PFS and OS	Bortezomib sensitivity	[127]
	miR-194	BM	UP	N = 44		Longer OS		[128]
	miR-223-3p	BM	DOWN	N = 94		Shorter OS		[129]
	miR-410	BM	UP	N = 97		Shorter PFS and OS		[130]
	miR-15a	BM	DOWN	N = 117		Shorter PFS and OS		[131]
	miR-16-1				MM detection			
	let-7b and miR-18a	Exosomes	DOWN	N = 156		Shorter PFS and OS		[95]
	miR-17 and miR-885-5p	BM	UP	N = 163		Risk stratification		[132]
	miR-720 and miR-1246	PB	UP	N = 60		Shorter PFS		[133]
	miR-15a-5p, miR-16-5p, miR-17-5p, and miR-20a-5p	Exosomes	DOWN	N = 330			Bortezomib resistance	[94]
	miR-15a, miR-16-1, miR-17, miR-20a, and miR-92-1	Plasma	UP	N = 85		Shorter PFS		[134]
	miR-153, miR-296, miR-490, miR-455, miR-500, and miR-642	BM	DOWN	N = 33		Shorter EFS		[135]
	miR-373, miR-548d, miR-554, and miR-888		UP					
	miR-4254	Serum, plasma **	UP	N = 627 ^‡^	Potential marker for MM			[136]
	miR-92a	Mainly serum **	UP	N = 1214 ^‡‡^		Shorter PFS and OS		[137]
	let-7e, miR-15a, miR-16, miR-25, and miR-744		DOWN					

* Data from the Cancer Genome Atlas (TCGA); ** Systemic review and meta-analysis of 32 ^‡^ and 10 ^‡‡^ relevant studies. Abbreviations: ALL, acute lymphoblastic leukemia; allo-HCST, allogeneic hematopoietic stem cell transplantation; AML, acute myeloid leukemia; BM, bone marrow; CLL, chronic lymphocytic leukemia; DLBCL, diffuse large B-cell lymphoma; EFS, event-free survival; CHT, chemotherapy; LN, lymph node; MDS, myelodysplastic syndrome; MM, multiple myeloma; MRD, minimal residual diseases; OS, overall survival; PB, peripheral blood; PFS, progression-free survival; RFS, relapse-free survival; UP/DOWN, up-/downregulation of miRNA expression compared to non-malignant controls.
